# Gene Expression Profiles in Stage I Uterine Serous Carcinoma in Comparison to Grade 3 and Grade 1 Stage I Endometrioid Adenocarcinoma

**DOI:** 10.1371/journal.pone.0018066

**Published:** 2011-03-23

**Authors:** Paulette Mhawech-Fauceglia, Dan Wang, Joshua Kesterson, Susanna Syriac, Kimberly Clark, Peter J. Frederick, Shashikant Lele, Song Liu

**Affiliations:** 1 Department of Pathology, Roswell Park Cancer Institute, Buffalo, New York, United States of America; 2 Department of Biostatistics, Roswell Park Cancer Institute, Buffalo, New York, United States of America; 3 Department of Gynecology-Oncology Surgery, Roswell Park Cancer Institute, Buffalo, New York, United States of America; 4 Department of Cancer Genetics, Roswell Park Cancer Institute, Buffalo, New York, United States of America; Vanderbilt University Medical Center, United States of America

## Abstract

**Background:**

Endometrial cancer is the most common gynecologic malignancy in the developed countries. Clinical studies have shown that early stage uterine serous carcinoma (USC) has outcomes similar to early stage high grade endometrioid adenocarcinoma (EAC-G3) than to early stage low grade endometrioid adenocarcinoma (EAC-G1). However, little is known about the origin of these different clinical outcomes. This study applied the whole genome expression profiling to explore the expression difference of stage I USC (n = 11) relative to stage I EAC-G3 (n = 11) and stage I EAC-G1 (n = 11), respectively.

**Methodology/Principal Finding:**

We found that the expression difference between USC and EAC-G3, as measured by the number of differentially expressed genes (DEGs), is consistently less than that found between USC and EAC-G1. Pathway enrichment analyses suggested that DEGs specific to USC vs. EAC-G3 are enriched for genes involved in signaling transduction, while DEGs specific to USC vs. EAC-G1 are enriched for genes involved in cell cycle. Gene expression differences for selected DEGs are confirmed by quantitative RT-PCR with a high validation rate.

**Conclusion:**

This data, although preliminary, indicates that stage I USC is genetically similar to stage I EAC-G3 compared to stage I EAC-G1. DEGs identified from this study might provide an insight in to the potential mechanisms that influence the clinical outcome differences between endometrial cancer subtypes. They might also have potential prognostic and therapeutic impacts on patients diagnosed with uterine cancer.

## Introduction

Endometrial carcinoma is the most common gynecologic malignancy in the United States [1]. Endometrioid adencoarcinomas (EAC) account for more than 80% of cases, and they tend to present as low grade, early stage tumors with good outcomes. While uterine serous carcinomas (USC) represent a minority (3–10%) of total endometrial cancer cases, they are usually high grade tumors with deep myometrial invasion, lymphovascular involvement, and a more aggressive clinical course [2]. USC is responsible for a disproportionate number of deaths reportedly due to the fact that most of these tumors have already spread outside the corpus. The 5-year survival rate for stage I-II EAC is estimated to range between 75–87%, and between 44–50% for stage I-II USC [3]–[5]. However, in EAC these outcomes vary with FIGO (International Federation of Gynecology and Obstetrics) tumor grade where five-year survival is 94% for FIGO grade 1 (EAC-G1) and 72% in high FIGO grade (EAC-G3) [6]. Several reports have suggested that women with early stages USC and clear cell carcinoma have a reasonably good 5- year disease free survival rate of 72%, which was comparable to that of early stage EAC- G3 tumors (76%) [7], [8], [9]. Furthermore, they even have similar 5-year vaginal-pelvic control rate with 97% for the early stages USC and clear cell carcinoma compared to 90% for early stage EAC- G3 tumors.

Even though numerous studies have reviewed the clinical outcomes of early stage USC when comparing EAC-G3 and EAC-G1, the gene expression profiles defining these tumor subtypes and the relations to their clinical behavior still remain unresolved. To unravel this mystery, we performed a genome-wide expression profiling analysis using the human Illumina bead microarray on 11 USC cases and compared them to 11 cases of stage I EAC-G3 and 11 cases of stage I EAC-G1, respectively. We found that the expression difference between USC and EAC-G3, as measured by the number of genes with significantly differential expression, is consistently fewer than that found between USC and EAC-G1. Pathway function annotation analyses suggested that the composition of enriched function terms was different among the two differentially expressed gene (DEG) sets. Gene expression differences for selected DEGs were subsequently confirmed by quantitative RT-PCR with a high validation rate. Our results, while preliminary, suggest that the more similar clinical outcome between stage I USC and stage I EAC-G3 potentially might be related to their inherent genetic similarity.

## Results

### Analysis of Microarray data

**Figure 1:**
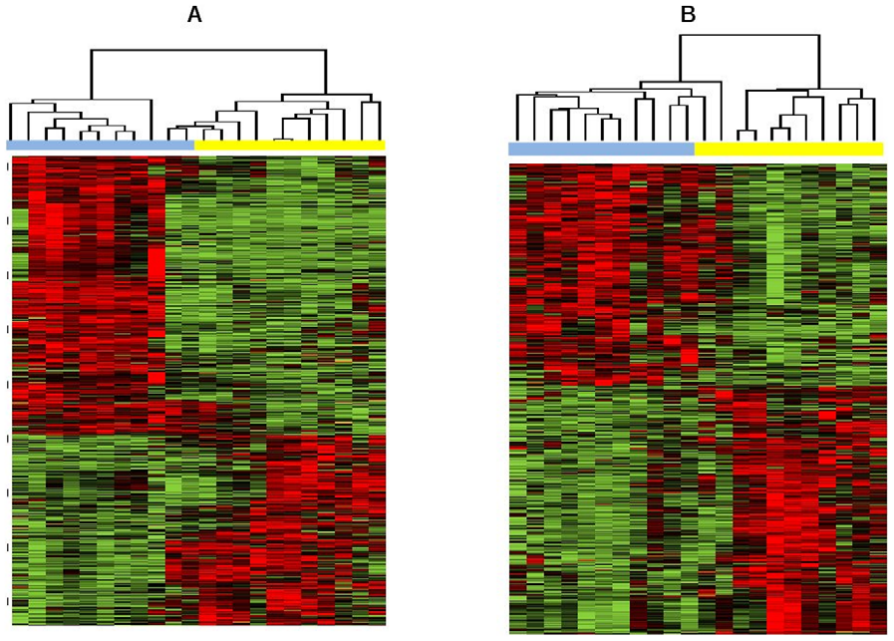
Hierarchical clustering of patient samples based on differentially expressed genes with at least 1.5-fold change obtained from USC versus EAC-G1 and USC versus EAC-G3, respectively. In clustering heat map, red means up-regulated while green means down regulated. *A) USC versus EAC-G1:* In clustering dendrogram, blue stands for USC samples while yellow stands for EAC-G1 samples. *B) USC versus EAC-G3:* In clustering dendrogram, blue stands for USC samples while yellow stands for EAC-G3 samples.

We performed two separate comparisons; USC *vs.* EAC-G1 and USC *vs.* EAC-G3, respectively. Hierarchical clustering of patients samples based on differentially expressed genes (DEGs) showed that the obtained DEGs are able to classify patients into their corresponding cancer subtype groups. As an example, the clustering results for DEGs with p<0.01 and at least 1.5 fold change are shown in **Figure 1** (*A: USC vs. EAC-G1*; *B: USC vs. EAC-G3*). The clear clustering results indicate that a direct comparison of the lists of DEGs identified here might help to explore the underlying mechanism accounting for clinical outcome differences between USC *vs.* EAC-G1 and USC *vs.* EAC-G3.

**Figure 2:**
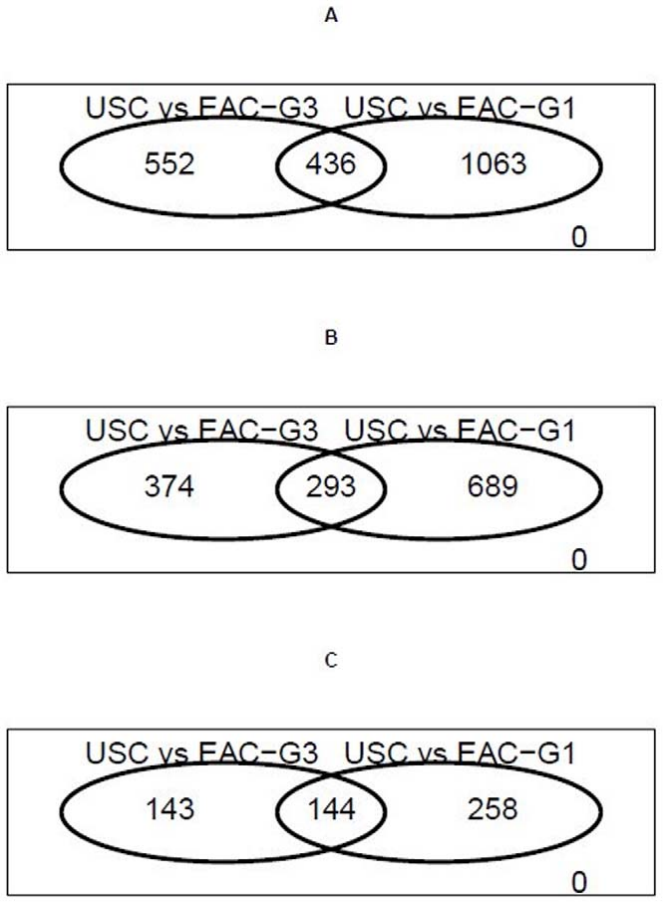
Venn diagrams showing the number of differentially expressed genes (DEGs) derived from USC *vs.* EAC-G3 comparison is consistently less than that derived from USC *vs.* EAC-G1 comparison. A) DEGs as defined by P-value <0.01. B) DEGs with at least 1.5-fold change. C) DEGs with at least 2-fold change.

We found that the expression difference between USC and EAC-G3, as quantified by the number of DEGs, was consistently less than that between USC and EAC-G1 (**Table 1** and **Figure 2**). At a significance cutoff of p<0.01 with at least 1.5 fold change, the comparison between USC and EAC-G3 could identify 667 DEGs (352 up-regulated and 315 down-regulated). Using the same significance criteria, the comparison between USC and EAC-G1 could identify 982 DEGs (398 up-regulated and 584 down-regulated). A total of 293 DEGs were shared by the two comparisons, resulting in 374 genes specific to USC *vs.* EAC-G3 and 689 genes specific to USC *vs.* EAC-G1. Similar trends were obtained when fold-change criteria was relaxed (*i.e.*, P<0.01 only) or more stringent (*i.e.*, P<0.01 & at least two-fold changes). The complete list of DEGs from the two separate comparisons described above is listed in **Table S2, S3**.

**Table 1:** Summary of the number of differentially expressed genes obtained from comparisons in USC *vs.* EAC-G1 and USC *vs.* EAC-G3, respectively.

	USC vs. EAC-G3	USC vs. EAC-G1	Overlap
**P-value<0.01**	988	1499	436
**UP**	509	639	211
**Down**	479	860	225
**P-value<0.01 & 1.5 FC**	667	982	293
**UP**	352	398	132
**Down**	315	584	161
**P-value<0.01 & 2 FC**	287	402	144
**UP**	142	137	52
**Down**	145	265	92

**Figure 3:**
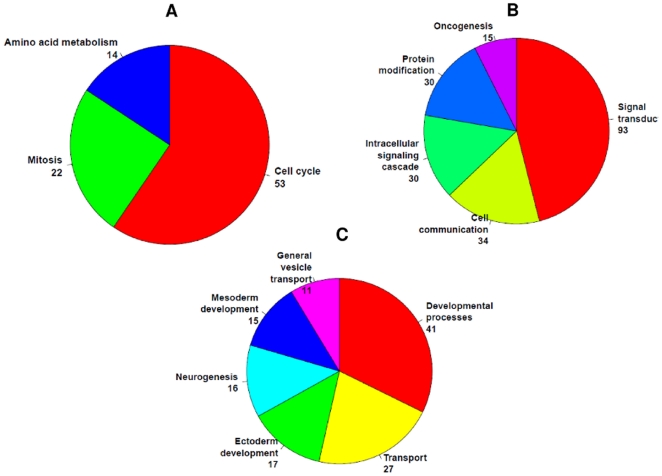
Enriched function terms for differentially expressed genes (DEGs) with at least 1.5-fold change identified by microarray. Function enrichment analyses are conducted using NCBI DAVID API server. The number following each enriched functional term is the number of annotated DEGs. *A)* Enriched functional annotation for DEGs specific to USC vs. EAC-G1 comparison. *B)* Enriched functional annotation for DEGs specific to USC vs. EAC-G3 comparison. *C)* Enriched functional annotation for DEGs shared by USC vs. EAC-G1 and USC vs. EAC-G3 comparison.

Further functional enrichment analysis showed that the composition of enriched function terms was different between the DEGs specific to USC vs. EAC-G1 and the DEGs specific to USC vs. EAC-G3 (**Figure 3**). For the 374 DEGs specific to USC vs. EAC-G3, the most enriched function terms are signal transduction, cell communication and oncogenesis. The most enriched function terms for the 689 DEGs specific to USC vs. EAC-G1 are cell cycle, mitosis and amino acid metabolism. For the rest of 293 DEGs shared by the two comparisons, the enriched function terms include transport, development process and neurogenesis. This suggests that different pathways or different components of common pathways might potentially account for the clinical outcome difference between USC *vs.* EAC-G1 and USC *vs.* EAC. However, these results are exploratory in nature and should be interpreted with cautions. Future studies with a larger sample size followed by functional experiments will be needed for validations.

### P53 and PTEN signaling pathway

Previous studies have shown that aberrant P53 and/or PTEN signaling pathways might play an important role in uterine and endometrial cancer. Therefore, we tested the hypothesis whether these two signaling pathway were significantly dysregulated in each of the two comparisons we performed. Briefly, the 13,863 genes were ranked based on the level of expression fold change (similar results are obtained based on t statistics score) for comparing USC *vs.* EAC-G1 (or USC *vs.* EAC-G3). This ranked list was used to analyze whether the fold change of genes for the P53 (or PTEN) signaling pathway were significantly deviated from those for the rest of genes. Statistical significance was estimated by *Kolmogorov–Smirnov* test [10].

**Figure 4:**
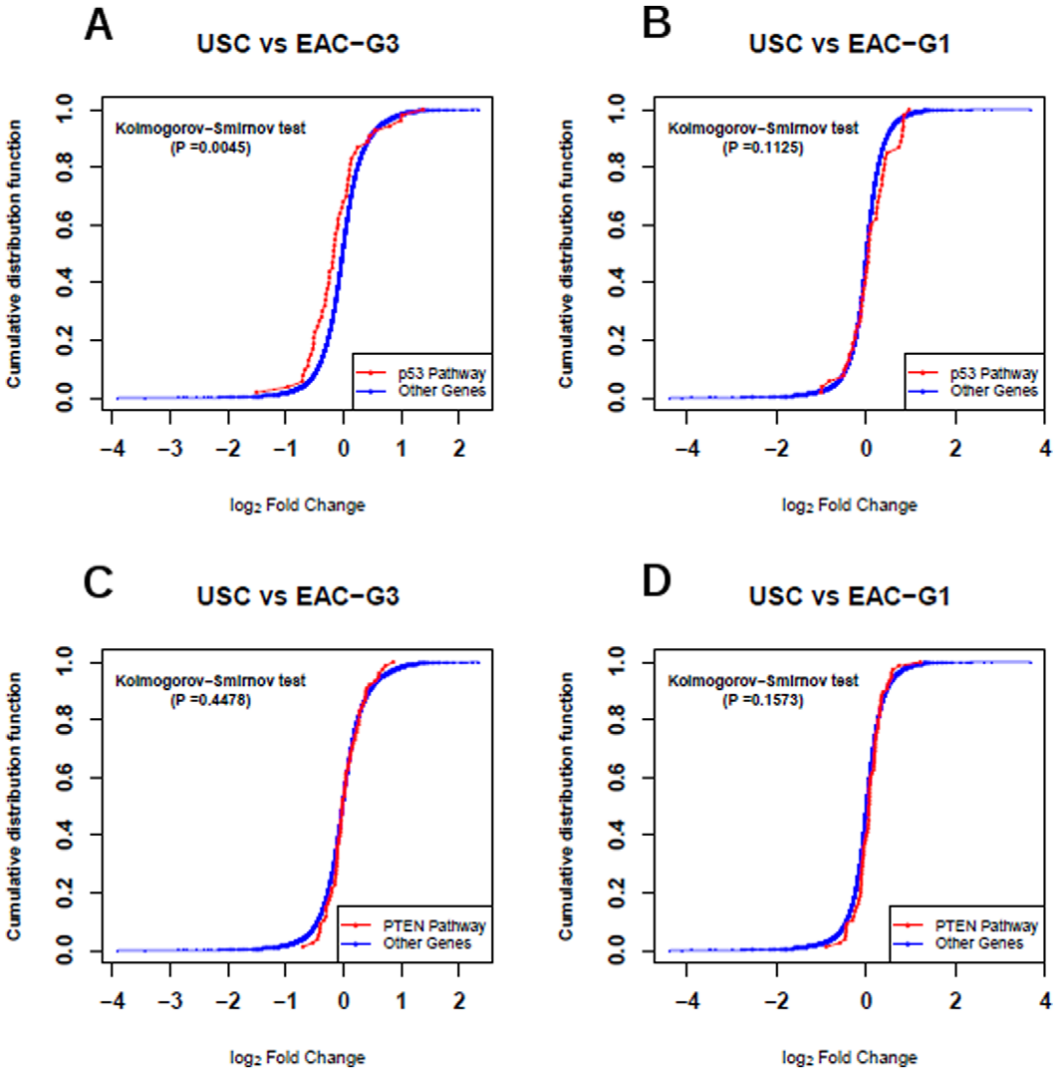
Log2 fold change of microarray estimated expression for genes from an investigated pathway are plotted as a cumulative distribution function (in red color) and compared with the corresponding cumulative distribution function for the rest of genes (i.e., not belonging to the investigated pathway, in blue color). A) P53 signaling pathway in USC vs. EAC-G3 comparison. The two cumulative distribution functions are significantly different (P = 4.5e-3) by the Kolmogorov-Smirnov test. B) P53 signaling pathway in USC vs. EAC-G1 comparison. C) PTEN signaling pathway in USC vs. EAC-G3 comparison. D) PTEN signaling pathway in USC vs. EAC-G1 comparison.

P53 gene was down-regulated (*Fold Change = -1.63, P = 2.99e-2*) in USC vs. EAC-G3 comparison, and P53 signaling pathway was significantly down-regulated in USC vs. EAC-G3 comparison (*K-S Pvalue = 4.5e-3*, Figure 4a). On the other hand, P53 gene showed no change (*Fold Change = −1.01*, *P = 0.97*) in USC vs. EAC-G1 comparison, and P53 signaling pathway was not significantly dysregulated in USC vs. EAC-G1 comparison (*K-S Pvalue = 0.11*, Figure 4b).

PTEN gene showed no change (*Fold Change = 1.05*, *P = 0.74*) in USC vs. EAC-G3 comparison, but are significantly up-regulated (*Fold Change = 1.67*, *P = 189e-3*) in USC vs. EAC-G1 comparison. However, PTEN signaling pathway was not found to be significantly dysregulated in either USC vs. EAC-G3 comparison (*K-S Pvalue = 0.45*, Figure 4c) or USC vs. EAC-G1 comparison (*K-S Pvalue = 0.16*, Figure 4d).

### Statistical estimation of false discovery rate

We used the approach of Benjamin and Hochberg [11] to estimate false discovery rate for the derived DEG lists (at a significance cutoff of p<0.01 with at least 1.5-fold change). For a conservative estimation based on all 13,863 genes passing the quality filter *(i.e.,* controlling on 13,863 statistical tests), the maximum FDR for the 667 DEGs derived from USC vs. EGC-G3 comparison is 9%, and the maximum FDR for the 982 DEGs derived from USC vs. EGC-G1 comparison is 14%. Hence, the FDR of both DEG sets are estimated to be <15%.

As we only considered DEGs with at least 1.5-fold change for further analysis, genes with less than 1.5-fold change can be excluded before statistics testing. The number of genes with at least 1.5-fold change is less than 1,750 for either USC vs. EGC-G3 or USC vs. EGC-G1 comparison. Controlled on the genes with at least 1.5-fold change *(i.e.,* applying fold-change analysis before statistics tests), the maximum FDR for DEGs (p<0.01 &FC>1.5) derived from USC vs. EGC-G3 comparison is 4.1%, and the maximum FDR for DEGs (p<0.01 &FC>1.5) derived from USC vs. EGC-G1 comparison is 2.5%.

### Quantitative -RT-PCR validation of microarray data

**Figure 5:**
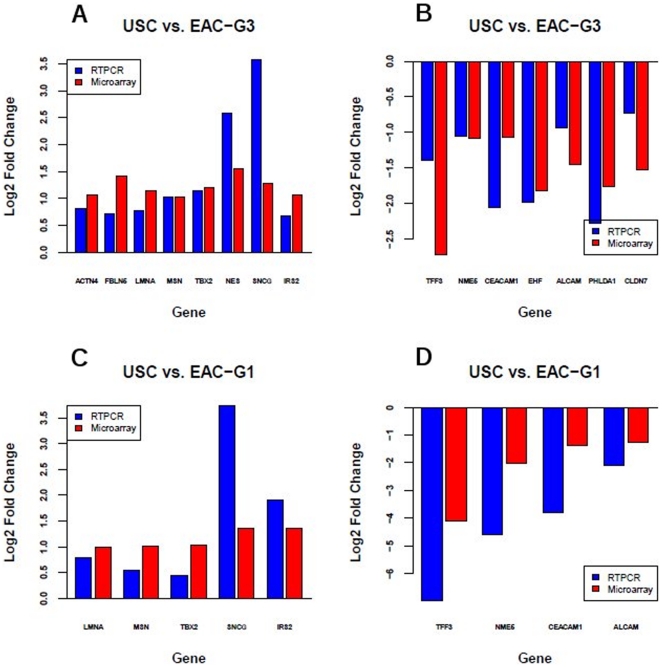
qRT-PCR validations of selected differentially expressed genes with at least 1.5-fold expression change identified by microarray. Blue bar is the fold change estimated by qRT-PCR, while red bar is the fold change estimated by microarray. The fold change is shown in log2 scale. A–B) For USC *vs.* EAC-G3, 15 of 15 selected genes showed at least 1.5-fold change in qPCR estimated expression levels and was concordant with the microarray data. C–D) For USC *vs.* EAC-G1, 7 of 9 selected genes showed at least 1.5-fold change in qPCR estimated expression levels and was concordant with the microarray data. The two genes with less than 1.5-fold change in expression level based on qPCR are MXN and TBX2.

We randomly selected 15 DEGs identified by microarray for validation by quantitative Real Time PCR (qRT-PCR). According to microarray analysis, all 15 selected genes are differentially expressed in USC *vs.* EAC-G3 with at least 1.5 fold changes. Selected genes include those up-regulated in USC *vs.* EAC-G3 (*FBLN5, NES, SNCG, ACTN4, MSN, LMNA, TBX2, IRS2)* and those down-regulated in USC *vs.* EAC-G3 (*CLDN7, EHF, NME5, ALCAM, PHLDA1, CEACAM1, TFF3).* qRT-PCR data of all 15 genes showed at least 1.5-fold change in expression levels in the USC vs. EAC-G3 comparison and were concordant with the microarray data, yielding a validation rate of 15/15 (**Figure 5a–b**). Microarray analysis showed that 9 of the 15 selected genes are also differentially expressed in USC *vs.* EAC-G1 comparison with at least 1.5 fold changes. These 9 genes include those up-regulated in USC *vs.* EAC-G1 *(SNCG, MSN, LMNA, TBX2, IRS2)*, and those down-regulated in USC *vs.* EAC-G1 *(NME5, ALCAM, CEACAM1, TFF3*). For USC *vs.* EAC-G1, 7 of 9 genes showed at least 1.5-fold changes in qRT-PCR estimated expression levels and were concordant with the microarray data, yielding a validation rate of 7/9 (**Figure 5c–d**). Even though *MSN* and *TBX2 genes* have less than a 1.5-fold change in expression level based on qRT-PCR in USC *vs.* EAC-G1 comparison, the direction (down or up-regulated) of their expression estimated by qRT-PCR are consistent with those estimated from microarray **(** **Fig 5c–d** **)**.

## Discussion

In this study, a genome-wide expression measurement was performed using the human Illumina bead microarrays on 11 cases each of stage I USC, EAC-G3 and EAC-G1. We performed two separate comparisons of USC *vs.* EAC-G1 and USC *vs.* EAC-G3, respectively, to determine differentially expressed genes (DEGs). A direct comparison of their gene expression patterns, as quantified by the number and overlap of DEGs, was performed to evaluate the differences among each category. Our results suggest that compared to USC vs. EAC-G1, USC vs. EAC-G3 was characterized by fewer DEGs, indicating that early stage USC is genetically more similar to early stage EAC-G3 than to early stage EAC-G1. Furthermore, we found that DEGs specific to USC vs. EAC-G3 are enriched for genes involved in signal transduction, while DEGs specific to USC vs. EAC-G1 are enriched for genes involved in cell cycle.

The function of DEGs derived from this study may provide insights into the molecular pathogenesis, and may explain the highly aggressive behavior of USC. In addition, these DEGs, once clinically verified in larger studies, may emerge as a potentially useful diagnostic and therapeutic marker. We provided detailed literature review about several DEGs observed in this study, including *SNCG*, *MSN* and *LMNA* which are up-regulated in USC, and *ALCAM*, *NME5* and *CEACAM1* which are down-regulated in USC.

Synuclein-gamma (*SNCG*) gene (also known as breast cancer-specific protein 1) was initially cloned from infiltrating breast carcinoma cells [12]. This gene is located at the 10q23.2 locus and highly expressed in several cancer types such as advanced stage of ovarian, breast, liver, prostate and colon cancer [13], [14]. Studies in breast cancer showed its over-expression can stimulate cell proliferation and induce invasion and metastasis [15]. Morgan *et al.* showed that *SNCG* was over-expressed in a uterine serous cancer cell line in comparison to endometrioid cancer cell line, and knockdown of *SNCG* in serous cancer cell line caused a significant decrease in cell proliferation and increased sensitivity to paclitaxel-induced apoptosis [16]. The up-regulation of SNCG in our series of USC patient samples reinforces that *SNCG* gene could be implicated in the pathogenesis of uterine serous tumors. However, to confirm SNCG as a prognostic marker and/or a therapeutic target in patients with endometrial cancer, future studies with larger sample size and functional experiments are needed.

*MSN* (membrane organizing extension spike protein) is closely related to the *EZR* and *RDX* proteins [17]. All three proteins constitute the ERM family which has been speculated to play a specific role in the coordination of signals that are required for tumor metastasis [18]. For example, *MSN* knockdown pancreatic cancer cells showed increased migration, invasion, metastasis and extracellular matrix organization [19], and *EZR* was found to be involved in the process of invasion of endometrial cancer cells [20], [21]. The high level of *MSN* mRNA in our series of USC samples suggests that it could have potential value as an indicator for aggressive endometrial cancer phenotype. Additional studies with larger group will be needed to evaluate its specific role.

Lamin A/C (*LMNA*) encodes type V filament protein which influences the activity of retinoblastoma protein and oncogenes including β-catenin. Consequently, the expression of *LMNA* is speculated to influence tumor progression [22]. *LMNA* was found to be expressed in colon cancer and its expression level positively associates with colon tumor progression and the risk of death [23], [24]. The role of *LMNA* up-regulation in our series of USC remained to be explored in future study.

*ALCAM* (Activated leukocyte cell adhesion molecule) is considered to be an important factor in epithelial cell integrity whose alternation can lead to invasion and even metastasis [25]. Decreased *ALCAM* expression levels were associated with aggressive behavior and poor outcome in several human tumors including breast cancer, pancreatic cancer and ovarian carcinoma [26], [27], [28]. The down-regulation of *ALCAM* in our series of USC samples reinforces its potential role as a molecular predictor of invasiveness and poor outcome in endometrial cancer patients.

*NME5* (non-metastatic cells 5, protein expressed in (nucleoside-diphosphate kinase)) gene is homologous to nm23-H1, a tumor suppressor gene whose expression is often lost in urinary bladder cancer and breast cancer [29], [30]. Carcinoembryonic antigen-related cell adhesion molecule1 (*CEACAM1*) has been reported to be implicated in tumor suppression and cell migration of bladder cancer [31], [32]. The prognostic value of down-regulation of *NME5* and *CEACAM1* observed in our USC samples remain to be established in future study.

One shortcoming of this study is the relatively small sample size which does not provide us enough power for statistical analysis of expression levels of DEGs and clinical characteristics. Our small-size study is exploratory in nature and the data should be interpreted with caution. Future large studies and functional experiments are necessary to confirm our observations and further explore the potential of DEGs to be utilized clinically as novel biomarkers for endometrial cancer.

## Materials and Methods

### Tissue specimens

Flesh-frozen cancer specimens were obtained from 33 patients undergoing surgery for uterine cancer at Roswell Park Cancer Institute (RPCI) including 11 cases of EAC-G1, 11 cases of EAC-G3 and 11 cases of USC. All cases were stage I disease. All of the tissue samples were collected under an Institutional Review Board-approved protocol at RPCI. Specimens were collected after written consent from the patient was obtained. The hematoxylin-eosin (HE) slides were reviewed by an expert gynecologic pathologist, to confirm the tumor subtype and FIGO grade. All patients were treated per National Comprehensive Cancer Network guidelines [33]. Patient charts were reviewed for postoperative follow-up, which ranged from 18 to 60 months. The detailed clinical and pathological information of patients are shown in **Table S1**.

### RNA preparation

The fresh frozen tissues were cut and examined to make certain that the tissue contained >80% tumor. Total RNA from 10–20 mg fresh frozen tissues were prepared using the RNeasy midi kits (Qiagen, Valencia, CA) following manufacturer's instructions. After elution, RNA samples were concentrated by EtOH precipitation at −20°C overnight, and resuspended in nuclease-free water. Before labeling, RNA samples were quantitated using a ND-1000 spectrophotometer (NanoDrop Wilmington, DE) and evaluated for degradation using a 2100 Bioanalyzer (Agilent Technologies, Santa Clara, CA). Samples were required to have a RIN>6.5, an OD 260∶280 of 1.9–2.1, an OD 260/230 >1.5 and >1.5 28S:18S ratio of the ribosomal bands for gene expression array analysis.

### Gene expression assay

Expression profiling was accomplished using the HumanHT-12 v3 whole-genome gene expression direct hybridization assay (Illumina, San Diego, CA) as previously published [34]. Each array contains full-length 50-mer probes representing more than 48,000 well-annotated RefSeq transcripts, including >25,400 unique, curated, and up-to-date genes derived from the National Center for Biotechnology Information Reference Sequence (NCBI RefSeq) database (Build 36.2, Release 22). Initially, 250 ng total RNA was converted to cDNA, followed by an in vitro transcription step to generate labeled cRNA using the Ambion Illumina Total Prep RNA Amplification Kit (Ambion, Austin, TX) as per manufacturer's instructions. The labeled probes were then mixed with hybridization reagents and hybridized overnight to the HumanHT-12 v3 BeadChips. Following washing and staining, the BeadChips were imaged using the Illumina BeadArray Reader to measure fluorescence intensity at each probe. The intensity of the signal corresponds to the quantity of the respective mRNA in the original sample. The expression profiles have been deposited in NCBI's Gene Expression Omnibus (GEO) with GSE accession number GSE24537.

### Data analysis

BeadChip data files are analyzed with Illumina's GenomeStudio gene expression module and R-based Bioconductor package to determine gene expression signal levels [35]. Briefly, the raw intensity of Illumina Human HT-12 v3.0 gene expression array was scanned and extracted using BeadScan, with the data corrected by background subtraction in GenomeStudio module. The *lumi* module in the R-based *Bioconductor* Package was used to transform the expression intensity into *log2* scale [36]. The log2 transformed intensity data were normalized using Quantile normalization function. For data quality control, we only kept the genes whose expression-detection *P-value* was ≤0.05 (*i.e.*, distinguishable from the background intensity) across >50% of samples in at least one group (USC, EAC-G1 or EAC-G3). A total of 13,863 genes passed this quality filtering for downstream analysis.

We then performed two separate comparisons for USC versus EAC-G1 and USC versus EAC-G3, respectively. We used the *Limma* program in the R-based *Bioconductor* package to calculate the level of gene differential expression for each comparison [37]. Briefly, for each comparison, a linear model was fit to the data (with cell means corresponding to the different cancer type and a random effect for array). For each comparison, we obtained the list of differentially expressed genes (DEGs) constrained by *Pvalue*<0.01.

Following single gene-based significance testing, we used the expression value of DEGs (*Pvalue*<0.01) with at least 1.5-fold change to cluster the patients. Our purpose was to determine whether the identified DEGs were able to serve as a gene signature to classify patients into their corresponding cancer type groups. Hierarchical clustering algorithm based on the average linkage of Pearson Correlation was employed [38]. The DEGs were analyzed for enriched biological process terms using the NCBI DAVID server (http://david.abcc.ncifcrf.gov) [39]. All calculations were carried out under R statistics computing.

### Quantitative real time PCR analysis

The expression of 15 genes *APLNR, CEBPA, CNTN1, ELF5, EPHA1, FBLN1, FOSB, FST, HOXD10, KIF14, LMO4, LPAR2, NME3, NR2F1, RASSF7, RHOBTB3, RPRM, TFF3* selected for validation was determined using Taqman quantitative RT-PCR (qRT-PCR) gene expression Assay On Demand Probe/Primers (Applied Biosystems, Foster City, CA), with housekeeping gene GAPDH as an endogenous control. Samples were run on the AB HT7900 Sequence Detection System according to default parameters, with three replicate assays for each gene in each sample. Using the RQ Manager Software 2.2.2 (AB, Foster City, CA) the data was analyzed with the baseline and the threshold verified for each gene of interest. qRT-PCR data were the normalized expression values in which the housekeeping gene GAPDH was used as the reference gene. For each assay, the average GAPDH Ct (Cycle threshold) value in the qPCR assay was subtracted from the Ct of gene of interest to obtain a ΔCt value (gene of interest - GAPDH).

## Supporting Information

Table S1 **The clinical and pathological information of patients.**
(XLS)

Table S2 **The list of differentially expressed genes obtained from comparison of USC** ***vs.*** **EAC-G1.**
(XLS)

Table S3 **The list of differentially expressed genes obtained from comparison of USC** ***vs.*** **EAC-G3.**
(XLS)
